# Protein kinase C modulates frequency of micturition and non-voiding contractions in the urinary bladder via neuronal and myogenic mechanisms

**DOI:** 10.1186/s12894-015-0030-9

**Published:** 2015-04-21

**Authors:** Joseph A Hypolite, Shaohua Chang, Alan J Wein, Samuel Chacko, Anna P Malykhina

**Affiliations:** 1 Division of Urology, Department of Surgery, University of Colorado Denver, Anschutz Medical Campus,12700 E 19th Ave, Mail Stop C317, Aurora, CO 80045 USA; 2 Department of Surgery, Cooper University, Camden, NJ USA; 3 Division of Urology, Department of Surgery, University of Pennsylvania, Philadelphia, PA USA

**Keywords:** Protein kinase C, Urinary bladder, Cystometry, Contractility

## Abstract

**Background:**

Protein Kinase C (PKC) dysfunction is implicated in a variety of smooth muscle disorders including detrusor overactivity associated with frequency and urgency of micturition. In this study, we aimed to evaluate the modulatory effects of endogenous PKC-dependent pathways on bladder storage and emptying function.

**Methods:**

We utilized *in vivo* cystometry and *in vitro* organ bath studies using isolated bladder muscle strips (BMS) from rats to measure contractility, intravesical pressure, and voided volume. Both *in vitro* and *in vivo* results were statistically analyzed using one-way repeated measures ANOVA between the groups followed by Bonferroni’s post-test, as appropriate (Systat Software Inc., San Jose, CA).

**Results:**

Effects of PKC activators, phorbol-12,13-dibutyrate (PDBu), and phorbol-12,13-myristate (PMA), were concentration-dependent, with high concentrations increasing frequency of micturition, and sensitivity of intramural nerves to electrical field stimulation (EFS), *in vitro,* while lower concentrations had no effect on BMS sensitivity to EFS. The PKC inhibitors, bisindolylmaleimide1 (Bim-1), (28 nM), and Ro318220 (50 μM) triggered an increase in the number of non-voiding contractions (NVC), and a decrease in the voided volume associated with reduced ability to maintain contractile force upon EFS, but did not affect peak force *in vitro.* Both low (50 nM) and high PDBu 1 micromolar (1uM) decreased the sensitivity of BMS to carbachol. Application of a low concentration of PDBu inhibited spontaneous contractions, *in vitro*, and Bim-1-induced NVC, and restored normal voiding frequency during urodynamic recordings *in vivo*.

**Conclusions:**

In summary, the effects of low PKC stimulation include inhibition of smooth muscle contractile responses, whereas high levels of PKC stimulation increased nerve-mediated contractions *in vitro,* and micturition contractions *in vivo.* These results indicate that endogenous PKC signaling displays a concentration-dependent contraction profile in the urinary bladder via both smooth muscle and nerve-mediated pathways.

## Background

Overactive bladder (OAB) is a complex clinical syndrome which affects approximately 17% of the US population, and is characterized by aberrant and involuntary contractions of the detrusor smooth muscle (DSM) [1-7]. The cause and underlying mechanisms of OAB have not been definitively elucidated, however, several hypotheses have been tested in animal models to understand the pathophysiology of OAB in humans. Among identified mechanisms are neurogenic and myogenic alterations of detrusor contractility, as well as changes in bladder stiffness, and ion channels associated with partial bladder outlet obstruction (PBOO), and other pathological conditions of the urinary bladder [3,8-15]. It was previously established that a number of DSM pathologies are linked to the changes in signaling proteins including, but not limited to, protein kinase C (PKC), Rho-associated kinase (ROK), and large conductance Ca^2+^-activated potassium (BK) channels [5,13,16-26]. Alterations in smooth muscle myosin isoforms from the fast phasic isoform to a more tonic one in PBOO [27,28] is correlated with aberrant calcium signaling [29], and higher resting levels of myosin light chain phosphorylation (MLCP) associated with bladder overactivity [13]. It has also been suggested that overactive bladder associated with hypertrophic changes in the smooth muscle may be due to a switch from M_3_ to M_2_ muscarinic receptor signaling which tends to show increased sensitivity [30-32]. Additionally, local changes in the bladder wall can affect central regulation of bladder function [33]. For instance, social stress in rats may induce bladder overactivity, frequency of micturition, and decreased voided volumes [34].

Protein kinase C dysfunction has been detected in a variety of smooth muscle disorders including the vasculature (high blood pressure) [17,35], trachea (asthma) [23,36,37], myometrium [23], and bladder (frequency of urination) [19]. Several studies also showed that PKC is involved in the modulation of neuronal activity [38,39], membrane receptors [40], and ion channels [22,41,42]. These wide range effects of PKC on the critical components of smooth muscle function suggest that alterations in any of these components may contribute to the pathophysiology of smooth muscle in the urinary bladder. In this regard, it was previously reported that down-regulation of PKC in PBOO-induced decompensated bladders is associated with frequency of urination, whereas in compensated bladders immunohistochemistry detected elevated PKC levels in the bladder tissue [19]. Pharmacologic inhibition of PKC in urothelium denuded BMS from normal rabbit bladders resulted in increased spontaneous contractions whereas activation of endogenous PKC activity suppressed these contractions [22]. Effects of PKC stimulation were concentration dependent: inhibition of spontaneous myogenic contractions and muscle tone was detected at low levels of endogenous PKC activation, while increased contractile force was observed at high levels of PKC activation. These data suggest that regulation of the detrusor tone and bladder contractile function within the micturition cycle may depend, at least in part, on a stimulus-dependent reciprocal mechanism mediated by PKC.

Bladder dysfunction often develops during progression of many non-urological diseases including neurodegenerative disorders, spinal cord injury, and diabetes [7,43,44]. A common theme among these conditions is that they affect the neural pathways innervating the lower urinary tract and usually trigger the development of neurogenic detrusor over activity. Damage to the central nervous system (CNS) inhibitory pathways and sensitization of peripheral afferents projecting to the bladder can also unmask primitive voiding reflexes leading to bladder over activity (reviewed in de Groat [10]). A number of previous [22,45] studies revealed that PKC can affect both nerve and muscle mechanisms that control bladder function [46]. Therefore, aberrant regulation of PKC due to pathophysiological changes may adversely affect the function of both nerves and muscle in a way that contributes to detrusor overactivity. In the present study, we performed pharmacological evaluation of urothelium intact BMS function *in vitro* (isolated muscle strips), and *in vivo* (cystometry) using PKC specific activators and inhibitors in order to determine their effects on nerve and muscle mechanisms underlying urinary bladder function. The data reveal that PKC displays a concentration-dependent activation profile in the bladder with low levels of stimulation inhibiting contractility, while high stimulation increases EFS-induced nerve-mediated, and micturition contractions.

## Methods

### Animals

Sprague-Dawley male rats (N = 32, 200-250 g, Charles River Laboratories, Malvern, PA) were utilized in this study. All protocols were approved by the University of Pennsylvania Institutional Animal Care and Use Committee. Rats scheduled to undergo *in vivo* cystometry were ordered pre-catheterized (urinary bladder) from the vendor and delivered three days post-surgery. The animals were kept in individual cages to avoid damage to the catheters by their cage mates. The animals were given 3 to 5 days after arrival for proper acclimation to the new environment, and relief of stress due to surgery and transportation.

### In vitro contractility studies

Rats were euthanized by an overdose of sodium pentobarbital (150 mg/kg), and the bladders were removed and placed into Tyrode’s buffer (124.9 mM NaCl, 2.5 mM KCl, 23.8 mM NaHCO_3_, 0.5 mM MgCl_2_, 0.4 mM NaH_2_PO_4_, 1.8 mM CaCl_2_, and 5.5 mM dextrose). Longitudinal urothelium intact BMS (~2 mm × 5 mm, 20-22 mg each, mucosa intact), were isolated and placed in individual organ baths (Radnoti, Monrovia, CA) containing 7 ml of Tyrode’s buffer equilibrated with 95% O_2_/5% CO_2_. One end of the strip was attached to a glass rod at the bottom of the organ chamber (Radnoti, Monrovia, CA) while the other end was attached to a force displacement transducer (Grass Instruments, Warwick, RI) connected to an AD Instruments power-lab computerized system (AD Instruments, Colorado Springs, CO). After 1 hour equilibration, the length of optimal force development (L_0_) was determined by manually increasing the length of each strip by 1.5 mm increments until maximal contractile force to electrical field stimulation at 32 Hz (EFS, 1 ms pulse width, 80 V pulse amplitude, 5 s stimulus duration) was achieved [22]. The bath solution was changed to fresh Tyrode’s buffer, and the muscle strips were allowed to equilibrate for 30 minutes in order to stabilize at L_0_ prior to performing the contractile studies.

### PDBu concentration-response curve

After initial tissue preparation as described above, increasing concentrations of a PKC activator, PDBu (20-640 nM), were applied to tissue strips to evaluate the effect of the drug on DSM tone.

### Carbachol concentration-response curve

Cumulative concentration-response curves were performed in the presence of both low (50 nM) and high (1 μM) PDBu, and Bim-1 (28 nM). PDBu treated muscle strips were first pre-incubated with the drug for 30 minutes, while Bim-1 treated muscle strips were pre-incubated for one hour prior to performing a concentration response curve. Control muscle strips received no treatment. After pre-incubation with PDBu, and Bim-1, a log-dose carbachol concentration-response curve was performed on all muscle strips (0.01-100 μM). PDBu and Bim-1 solutions were added to each bath solution reaching the appropriate final concentration in each organ bath.

### Frequency-response curve in response to EFS

After equilibration in Tyrode’s buffer as described above, individual muscle strips underwent an EFS protocol with ascending frequency of applied electric stimuli ranging from 0.5 to 32 Hz. Control muscle strips received no pharmacological treatment prior to stimulation. To evaluate the effects of PKC on detrusor contractility, strips were first incubated with either Ro318220, (4.5 μM-121.5 μM, PKC inhibitor, 1 h), bisindolylmaleimide-1 (Bim-1, PKC inhibitor, 4 nM-32 nM, 1 h), PMA (12.5 μM–100 μM, PKC activator, 30 min), or with phorbol-12,13-dibutyrate (PDBu, PKC activator, 10 nM-1 μM, 30 min), before running an EFS protocol. Stimulus-response curves were calculated in grams of tension per individual muscle strip.

### Cystometry studies

For urodynamic assessment of bladder function, conscious rats were placed in cystometry cages (24 cm length, 16 cm width, and 12 cm height) without restraint and allowed to acclimate for 30 min. The tip of the exteriorized bladder catheter located at the base of the rat neck, was connected to a pressure transducer and the infusion pump of the cystometry station (Small Animal Laboratory Cystometry, Catamount Research and Development, St. Albans, VT) using a T-shaped valve. Room temperature saline solution (0.9% NaCl) was infused into the bladder at a rate of 80 μl/min. Voided urine was collected in a tray connected to a force displacement transducer, which actively integrated results into the data acquisition system. Each animal was observed for approximately six to eight voiding cycles. For animals receiving Bim-1 (28 nM), and PDBu (50 nM-1 μM), PMA (50 μM), Ro318220 (50 μM), the concentrated drugs were initially diluted by saline to achieve the final concentrations, and then were infused in the urinary bladder in the same fashion as for control experiments. Urodynamic values were continuously recorded using data acquisition software (Small Animal Laboratory Cystometry, Catamount Research and Development). The following cystometric parameters were recorded and analysed in this study: bladder capacity, pressure at the start of micturition, micturition rate, continuous intra-vesical pressure, inter-micturition interval, and the number of non-micturition contractions. Non-micturition contractions were defined as increased values of detrusor pressure from the baseline that had amplitudes of at least a third of maximal pressure from the start of micturition.

### Statistical analysis

All data are expressed as the mean ± SEM. N reflects the number of animals and n is the number of strips/recordings. Contractility of DSM in organ bath studies was measured and analyzed using a computerized system for data acquisition and analysis (AD Instruments, Colorado Springs, CO). The peak force (PF,g) and integral force (IF, g/s) were calculated as following: the peak force (PF, g) is the maximal amplitude of contraction in response to respective stimulation measured from the baseline; and the integral force (IF, g/s) is the area under the time-force curve of a single contraction. Cystometric parameters were uploaded from the acquisition software into analysis software (SOF-552 Cystometry Data Analysis, Version 1.4, Catamount Research and Development Inc., St. Albans, Vermont). Maximum pressure at micturition, bladder capacity, micturition volume, number of non-micturition contractions, inter micturition interval, and micturition rate indices were calculated. Both *in vitro* and *in vivo* results were statistically analysed using one-way repeated measures ANOVA between the groups followed by Bonferroni’s post-test, as appropriate (Systat Software Inc., San Jose, CA). Differences between the groups were considered statistically significant at p ≤ 0.05.

## Results

### PKC activation reduces contractile force and BMS spontaneous activity at low concentrations and increases contractility force at higher concentrations

**Figure 1:**
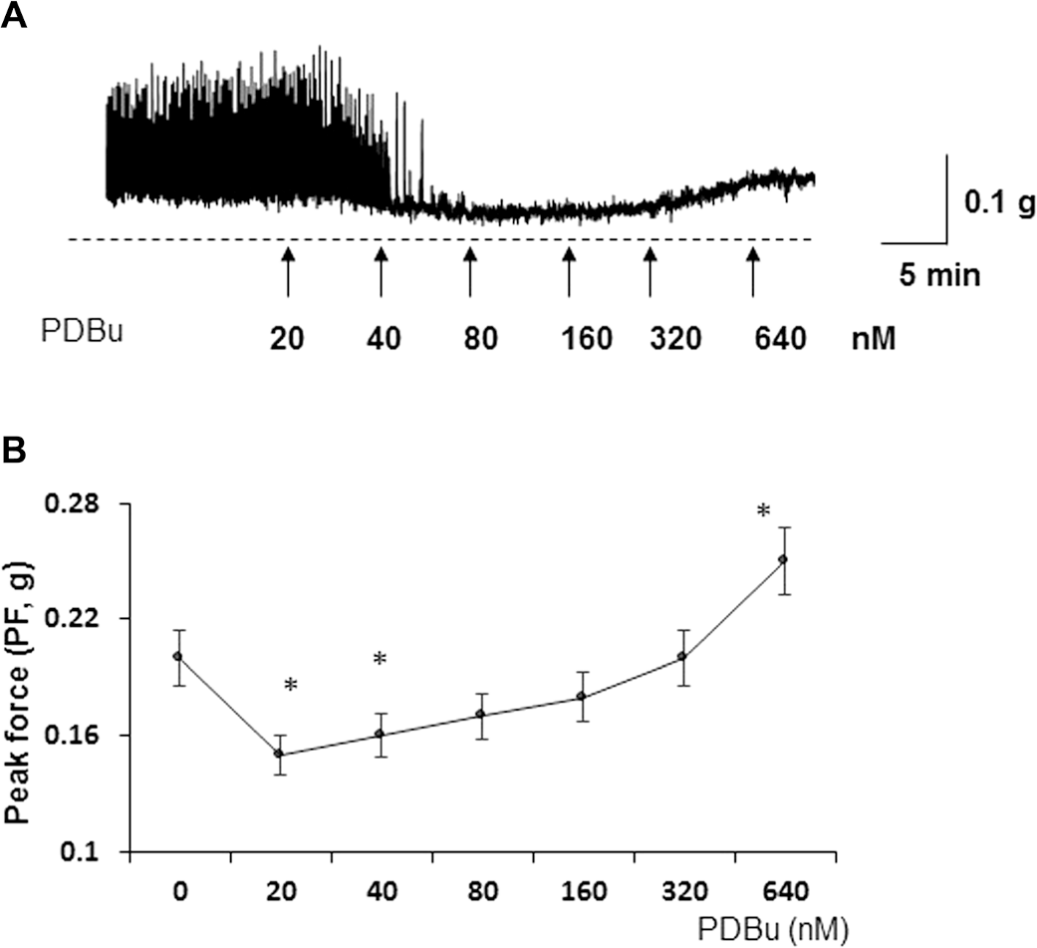
Concentration dependent effects of PKC stimulation by PDBu on spontaneous contractions of isolated detrusor strips. **A**, Spontaneous contractile activity of DSM strips upon increasing concentrations of PDBu (0–640 nM). At the initial concentration of 20 nM, PDBu inhibited spontaneous contractions, and caused a small but significant decrease in the baseline BSM tone. Continued application of PDBu reversed this decline and increased the muscle force above the initial basal levels. **B**, Summarized maximal amplitude of contractions indicates that PDBu significantly reduced PF generation (peak force, PF) at lower PDBu concentrations (20-40 nM; N = 5, n = 7 in each group, p < 0.05 to baseline).

The biphasic effects of PKC activation by PDBu on urothelium intact isolated bladder strips in response to increasing concentrations of the drug is displayed in Figure 1. This phenomenon has been previously reported in urothelium denuded BSM strips isolated from the rabbit urinary bladder, and confirmed that the urothelium did not play a role in PKC-induced inhibition of bladder contractions [22]. Present data reveal that at low levels of PKC activation (20 nM), PDBu inhibited spontaneous contractions, and lowered the basal smooth muscle tone from 0.2 ± 0.014 g to 0.15 ± 0.011 g tension, a 25% decrease (Figure 1 B, N = 5, n = 7, p ≤ 0.05 to baseline). Further exposure to PDBu up to a maximal concentration of 640 nM led to a reversal of the initial reaction and an increased muscle tone up to 0.25 ± 0.017 g of tension, a 25% increase above the initial basal level (N = 5, n = 7, p ≤ 0.05 to baseline).

### High level of PKC activation by PDBu enhances the maximal contractile force of BMS to EFS-induced nerve stimulation

**Figure 2:**
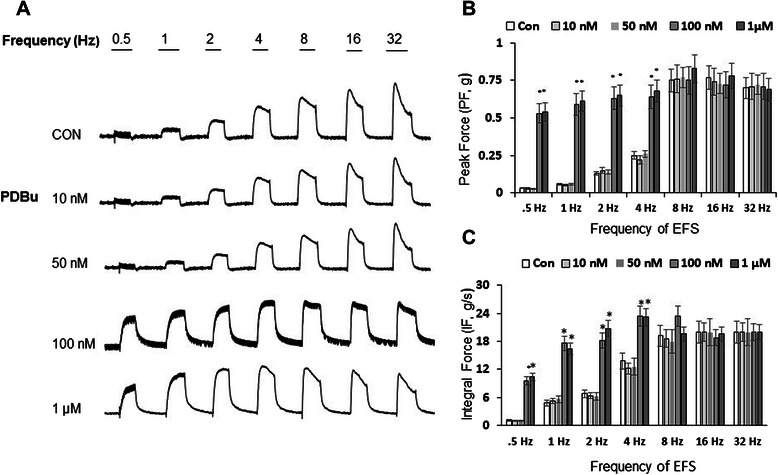
Effects of PDBu, a PKC activator, on contractility responses of BSM to EFS *in vitro*. Muscle strips were pre-incubated for 30 minutes with different concentrations of PDBu (0.01-1 μM), followed by a number of increasing frequencies of EFS stimulations (0.5-32 Hz). **A**, Representative raw tracings for control (upper panel), and experimental (lower panels) groups treated with PDBu. **B**, Summarized peak amplitude of contractions (PF, N = 5, n = 8 for each concentration; p ≤ 0.05 at stimulations up to 4 Hz). **C**, PDBu caused a significant increase in IF at 0.5-4 Hz (N = 5,n = 8, p ≤ 0.05 to control values). The results were not significantly different at 8, 16, and 32 Hz for PF and IF.

Our preliminary studies indicated that PKC activation by PDBu had a concentration-dependent effect on EFS-induced nerve stimulation in isolated BMS. High (1 μM) concentration of PDBu amplified both the peak force (PF) response, and integral force (IF) of the bladder strips at low levels of EFS (0-4 Hz). We, therefore, followed the changes in BMS contractility at different concentrations of PDBu to determine the threshold for EFS-induced sensitivity in BMS. Our results (Figure 2) indicate that at a concentration of 100 nM and above, PDBu induced a significant increase in both PF, and IF in BMS from 0.5 to 4 Hz of EFS. At 4 Hz of EFS, the PF response in the presence of 100 nM PDBu was already 98 ± 11% of maximal force generation (measured at 32 Hz), and 117 ± 13% of maximum value for IF (Figure 2 B and C, N = 5, n = 8, p ≤ 0.05 for both parameters). By comparison to PDBu, the control bladder strip developed only 52 ± 6% of PF at 4 Hz, and 69 ± 8% of maximum value for IF. The greatest difference was seen at 0.5 Hz with 100 nM PDBu treatment. These data confirm the concentration-dependent effects of the PKC activator, PDBu on EFS-mediated contractility of isolated BMS.

### PKC activator, PMA, enhances the contractile force response to EFS in BMS

**Figure 3:**
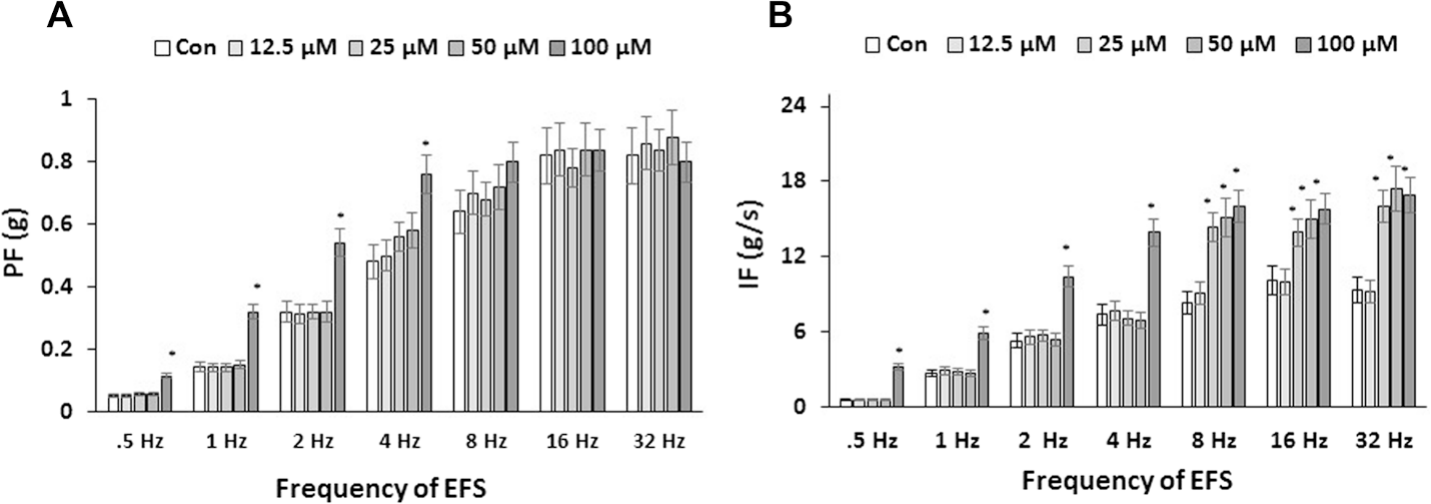
Concentration-dependent effects of alternative PKC activator (PMA) on EFS-induced contractility. **A**, Summarized maximal amplitude of contractions indicates that PMA (100 μM) significantly enhanced PF generation at 0.5-4 Hz (N = 4, n = 6 in each group, p ≤ 0.05 to baseline). **B**, Application of PMA (25 μM and above) also increased IF of muscle strips at 8-32 Hz (N = 4, n = 6 in each group, p ≤ 0.05 to control values).

In order to provide additional evidence for our abovementioned findings using PDBu, we used a similar PKC activator, PMA, to evaluate its effect on EFS-induced contractions. The data from this experimental set showed that PMA had significant effects on both PF and IF but required much higher concentrations to be effective. Figure 3 (A and B) present summarized data confirming that PMA (100 μM) stimulated an increase in PF and IF up to 4 Hz of EFS in comparison with control strips not treated with PMA. Similar to PDBu results, the greatest change was observed at lower frequencies (0.5-2Hz), where the PF increased by 228 ± 20.9%, 228 ± 21.5%, and 168 ± 16.2% (Figure 3 A), and the IF increased by 561 ± 52.3%, 219 ± 21.9%, and 196 ± 20.2%, respectively (Figure 3 B, N = 4, n = 6 in each group, p ≤ 0.05 to control values). Starting from 25 μM of PMA, and at 8-32 Hz of EFS stimulation, the drug caused an average increase in IF of 82 ± 7.9%, 48 ± 5.4%, and 80 ± 9.3% for 8, 16, and 32 Hz of EFS, respectively (Figure 3 B, N = 4, n = 6 in each group, p ≤ 0.05 to control values).

### Inhibition of PKC with Bim-1 reduces the ability of BMS to maintain muscle force

**Figure 4:**
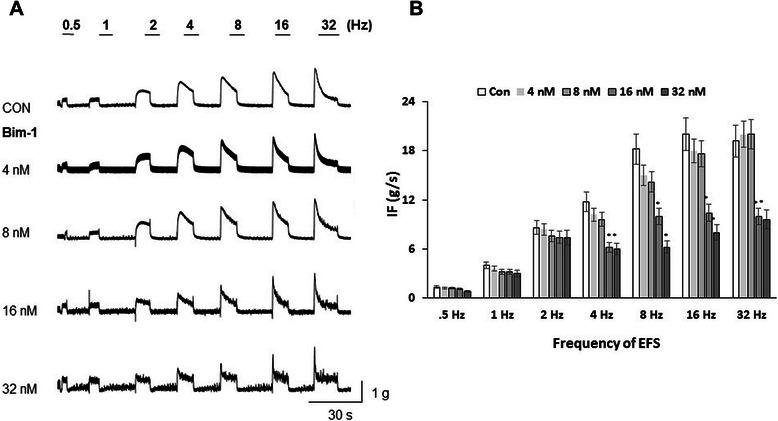
Contractile responses of the detrusor muscle strips to EFS upon application of PKC inhibitor, Bim-1. Muscle strips were pre-incubated with different concentrations of Bim-1 (4-32 nM) for 60 min prior to EFS-induced nerve stimulation. **A**, The upper panel shows the contractile tracers of control strips at tested frequencies, and the lower panels represent BSM contractility after Bim-1 treatments. EFS was carried out at 0.5-32 Hz. **B**, Summarized integral force (IF, g/s, n = 5 for each group) of BSM responses to Bim-1. Incubation with Bim-1 caused a significant reduction in IF upon 4 Hz and higher frequencies of EFS-induced contraction (N = 3, n = 5, p ≤ 0.05 to control for 16-32 μM of Bim-1).

Previous studies established that down-regulation of PKC in PBOO is associated with frequency of urination and a decreased volume of voided urine [19]. Additionally, Bim-1, a specific inhibitor of PKC, did not affect the PF at 32 Hz but reduced IF (ability to maintain muscle force) by ~50% in isolated BMS during *in vitro* studies when compared with control strips [22]. This observation suggested that Bim-1 induced a significant loss of smooth muscle ability to maintain force which is a requirement for physiological bladder emptying. We, therefore, evaluated BMS force generation over a range of EFS frequencies in the presence and absence of Bim-1, at different concentrations, to determine if this finding was dependent on the frequency of stimulation (Figure 4). Our data did not determine a significant difference between PF with and without Bim-1 over a range of applied frequencies (0.5-32 Hz, Figure 4 A) and concentrations of Bim-1 (4 nM- 32 nM). There was no substantial difference between IF values of strips with or without Bim-1 treatment at lower frequencies of EFS (0.5-2 Hz) either (Figure 4 B). However, EFS with frequencies above 2 Hz caused a significant reduction in IF of BMS subjected to Bim-1 (16 and 32 nM) in comparison with control group (Figure 4 B). The IF declined significantly at stimulation frequencies from 4 to 32 Hz at 16 and 32 nM of Bim-1. At a concentration of 16 nM Bim-1, the decline in IF from control values was 53 ± 5.1%, 55 ± 4.9%, 52 ± 5.2%, and 52 ± 4.8% at 4, 8, 16, and 32 Hz of EFS, respectively (N = 3, n = 5, p ≤ 0.05 to control group).

### PKC inhibitors reversed EFS-induced sensitivity of BMS upon high PKC stimulation, and decreased IF in bladder strips

**Figure 5:**
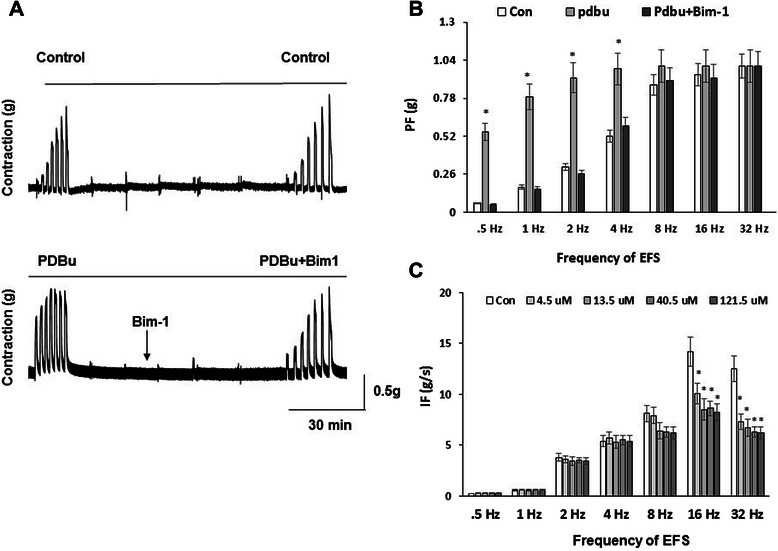
Increased sensitivity of SM to EFS during high PKC stimulation is reversed by pre-incubation with Bim-1. **A**, Experimental design included initial pre-incubation for 30 min with 100 nM of PDBu (bottom panel) followed by frequency response curve to EFS (0.5-32 Hz). Control strips received no PDBu (top panel). 20 minutes after the frequency response curve, Bim-1 (100 nM) was added for 45 minutes to the PDBu treated muscle strips. The frequency response curve was then repeated for both control and drug treated DSM strips. **B**, Analysis of the peak force shows that addition of Bim-1 to PDBu-treated strips caused a substantial reduction in PF which was similar with baseline levels at 0.5-4 Hz of EFS (N = 5, n = 7 in each group, p ≤ 0.05 to PDBu treatment at respective frequencies). There was no significant enhancement by PDBu, nor reversal by Bim-1, at 8, 16 and 32 Hz of stimulation. **C,** Ro318220, an alternative PKC inhibitor, significantly reduced the integral force of BSM contractions at both low (4.5-13.5 μM) and high (40.5-121.5 μM) concentrations upon 16 and 32 Hz of EFS (N = 4, n = 6 in each group, p ≤ 0.05 at 16 and 32 Hz to baseline).

In order to determine if increased sensitivity to EFS by high PDBu could be reversed by PKC inhibitors, we tested the effects of Bim-1 on muscle strips previously subjected to high concentration of PDBu. Figure 5 A (upper panel) shows the EFS-frequency response for the control strip without any treatment. Figure 5 A (lower panel) includes the effects of PDBu (100 nM) stimulation on EFS-dependent contractile responses alone, and after incubation with Bim-1 in the presence of PDBu. At the lowest frequency of EFS (0.5 Hz), PDBu increased the PF by 8-fold (N = 5, n = 6, p ≤ 0.05 to control); this effect was blocked by application of Bim-1 (Figure 5 B). At 0.5-4 Hz of EFS, the PF of PDBu-enhanced responses was reversed by addition of Bim-1, and the PF values were similar to the control group (Figure 5 B). PDBu had no significant effect on enhancement of PF at 8, 16 and 32 Hz of EFS, paralleling the absence of effect to Bim-1 stimulation at the same frequencies.

In order to provide further support for the effects of PKC inhibition on BMS contractility, we tested another PKC inhibitor, Ro318220 (Figure 5 C). Experiments with Ro318220 established that the drug significantly reduced IF of muscle strips, but required much higher concentrations in comparison with Bim-1 to be effective. Between 0.5 and 8 Hz of applied EFS, there was no significant difference in the level of IF at any concentration of Ro318220 (4.5-121.5 μM). However, at 16 and 32 Hz of EFS, Ro318220 caused a significant decrease in IF of the strips with the greatest effect observed at 40.5 μM and 121.5 μM (N = 5, n = 7, p ≤ 0.05 to control). At 40.5 and 121.5 μM, Ro318220 (at 16 Hz of EFS) decreased IF of BMS to 60 ± 7.2% and 58 ± 5.4% of control values (Figure 5 C, N = 4, n = 6, p ≤ 0.05).

### Effects of extracellular calcium on the contractile responses of isolated BMS

**Figure 6:**
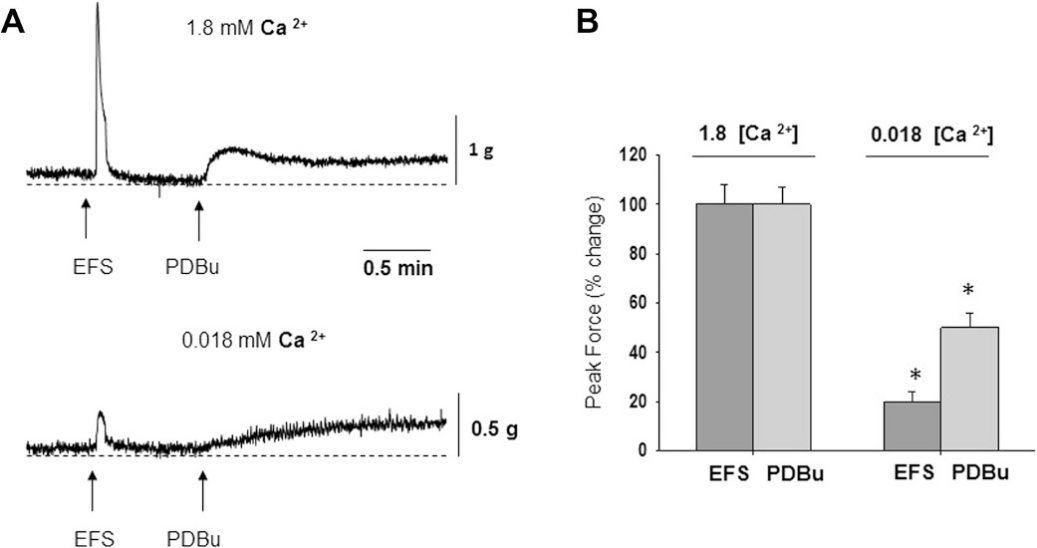
Effects of calcium concentration on EFS- and PDBu-induced force generation in isolated BSM strips. **A**, Isolated DSM strips were subjected to EFS (32 Hz) and PDBu (1.0 μM) in the presence of normal (1.8 mM, top trace) and very low (0.018 mM, bottom trace) Ca2+ concentration. **B**, Reduction of the calcium concentration by 100-fold resulted in a significant decrease in the contractile response of BSM strips to both EFS and PDBu.

Previous studies established that endogenous PKC activity is regulated by Ca^2+^-dependent and independent mechanisms [35,40,45,47]. Therefore, we recorded the contractile responses of BMS to EFS and PDBu in solutions with normal (1.8 mM) and very low (0.018 mM) calcium (Figure 6). A reduction in calcium concentration by 100-fold led to an 85% decrease in the response to EFS (32 Hz, Figure 6 A), and also caused a 50% decrease in the response to high concentration of PDBu (1.0 μM, Figure 6 B).

### Effects of a high concentration of PDBu on BMS frequency-contractility response upon EFS

**Figure 7:**
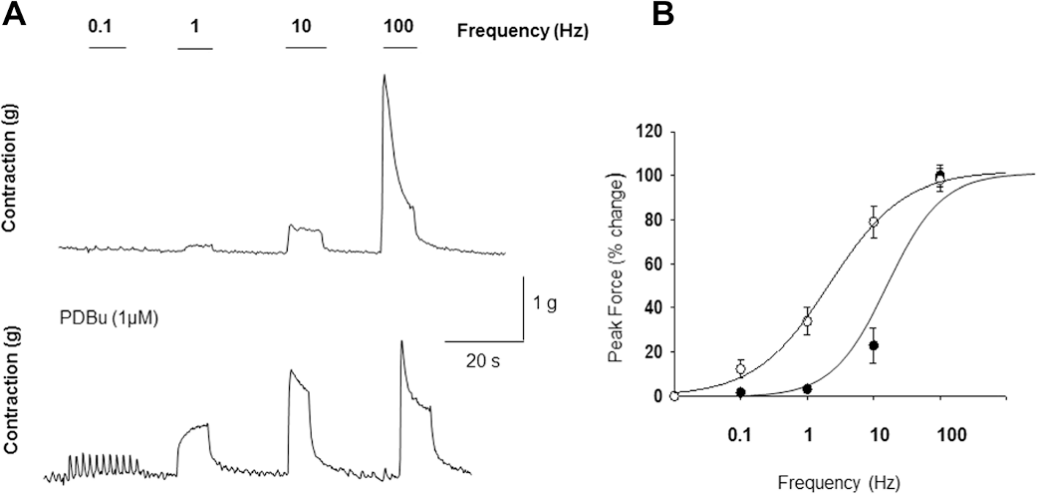
High level of PKC activation increases sensitivity of BSM strips to low frequency of EFS. **A**, Upper panel shows the response of the control strip to increasing frequencies of EFS whereas the lower panel represents the frequency-response curve after incubation with PDBu (1 μM). **B**, PDBu caused a significant leftward shift (open circles) in BSM sensitivity to EFS representative of a 5-fold increase in muscle responsiveness to EFS in the presence of high PDBu concentration (N = 5,n = 6, p ≤ 0.05).

Our prior data (Figure 2) confirmed an increased sensitivity to EFS upon high level of stimulation of PKC activity, however, low levels of PKC activation had no significant effect on BMS response to EFS stimulation. In order to determine the stimulation frequency that produced half-maximal response (EFS_50_), we obtained a logarithmic frequency-response curve (0.1–100 Hz) in the presence of high PDBu. EFS50 values were determined for each strip using a sigmoidal curve fit of the data (SigmaPlot 11 Software, Systat Software Inc, San Jose, CA). Figure 7 A (upper panel) shows the contractile response of control strips whereas the lower panel presents the raw traces of BMS subjected to PDBu application (1 μM). Figure 7 B presents the summary data of contractile responses of BMS treated with and without PDBu. Application of PKC activator caused a significant leftward shift of dose response curve with EFS_50_ value of 2.14 ± 0.14 Hz in comparison with control value of 10.3 ± 1.2 Hz (Figure 7 B). This is indicative of a 5-fold increase in sensitivity of BMS strips to EFS incubated with a PKC activator (N = 5, n = 6, p ≤ 0.05 to control group).

### Application of a PKC activator decreases BMS sensitivity to carbachol

**Figure 8:**
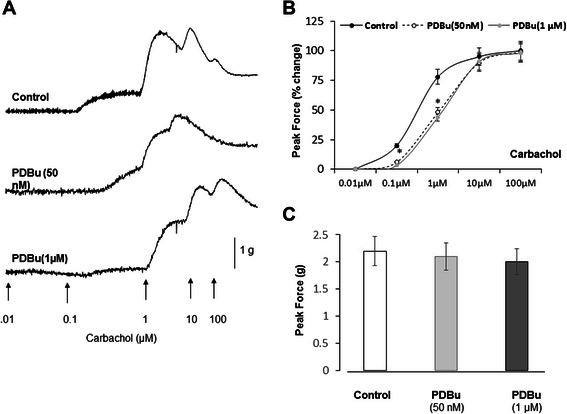
Both low and high PKC stimulation reduces sensitivity of BSM strips to stimulation of muscarinic receptors by carbachol. **A**, Raw tracers of contractile responses to increasing concentrations of carbachol in the control (top panel) isolated BSM strip, and strips treated with low (middle panel, 50 nM) and high (bottom panel, 1 μM) concentration of PDBu. **B**, Both low and high PDBu caused a significant rightward shift (reduced sensitivity) in the carbachol concentration response curve. The EC50 values were 0.56 ± 0.049, 4.2 ± 0.35, and 4.7 ± 0.47 nM for control (black circles), PDBu (50 nM, open circles, N = 4, n = 6, p ≤ 0.05 to control), and PDBu (1 micromolar (1uM), grey circles p ≤ 0.05 to control)), respectively. **C**, Despite the reduced sensitivity of BSM strips to carbachol stimulation after PDBu treatment, there was no significant difference in the PF of contractions between the groups. The PF values were 2.2 ± 0.25, 2.1 ± 0.24, and 2.0 ± 0.18 g for control, PDBu (50 nM), and PDBu (100 nM) groups, respectively.

It was previously shown that PDBu can inhibit spontaneous myogenic contractions *in vitro* at low levels of PKC activation. Our cystometric experiments also confirmed that the PKC inhibitors, Bim-1 and Ro318220, can induce frequency and NVC, and that PDBu can reverse Bim-1-induced frequency (please see below). We, therefore, tested the effects of different concentrations of PDBu on cholinergic sensitivity in DSM. As seen in Figure 8A, PDBu at 50 nM and 1 μM caused a significant rightward shift in the carbachol concentration response curve when compared to control group (Figure 8 B, N = 4 and n = 6 in each group, p ≤ 0.05). The EC_50_ values were 0.56 ± 0.049 nM, 4.2 ± 0.35 nM, and 4.7 ± 0.47 nM for control, PDBu (50 nM), and PDBu (1 μM) groups, respectively. This represented an approximate 8-fold decrease in sensitivity of BMS to carbachol after PDBu application. There was no effect on the maximum force produced with or without PDBu application.

### Bim-1 does not affect carbachol concentration-response curve

**Figure 9:**
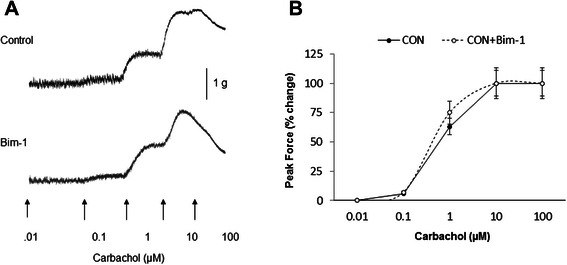
Effects of PKC inhibition by Bim-1 on carbachol dose-response curve. **A**, Raw tracers of contractile responses to increasing concentrations of carbachol in the control (top panel) strips, and strips pre-treated with Bim-1 (bottom panel). **B**, Bim-1 (28 nM) had no significant effect on either the sensitivity to muscarinic stimulation (EC 50), or the maximum contraction (PF) upon carbachol application (p = 0.061 for all concentrations of carbachol).

Figure 9 A shows the representative raw data for control (upper trace), and Bim-1-treated (lower trace) muscle strips upon stimulation of muscarinic receptors with carbachol. The summary data in Figure 9 B reveal that Bim-1 had no significant effect on sensitivity to carbachol stimulation, nor on the PF generation in comparison with control (EC_50_ = 0.52 ± 0.06 μM for control group (N = 4, n = 7) *vs* EC_50_ = 0.54 ± 0.06 μM for Bim-1 group (N = 5, n = 8, p ≤ 0.05 to control).

### Effects of PKC activation by PMA on urinary bladder function in vivo evaluated by cystometry

**Figure 10:**
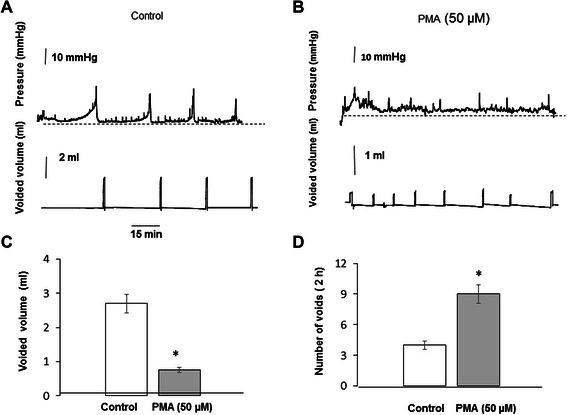
Cystometric evaluation of bladder function upon intravesical instillation of a PKC activator, PMA. **A**, Representative traces of intravesical pressure (top panel) and voided volume (lower panel) in rats instilled with normal saline. **B**, Representative traces of intravesical pressure (top panel) and voided volume (lower panel) in rats instilled with high concentration of PMA (50 μM). The rate of infusion was 80 μl/min. C, Perfusion of the urinary bladder *in vivo* with high PMA induced a significant increase in the frequency of micturition contractions, and a simultaneous decrease in the voided volume. D, Intravesical instillation of PMA also increased voiding frequency from 4 ± 0.5 to 8 ± 0.9 voids over the 2 h period (N = 4 in each group, p ≤ 0.05 to control).

In addition to *in vitro* studies, we carried out similar experiments utilizing *in vivo* urodynamic approach in awake rats. We tested the effects of PMA, a PKC activator, on bladder function. Intravesical instillation of PMA (50 μM) caused a significant increase in frequency of micturition contractions, and decline in the voided volumes when compared to the control. Figure 10 A shows the representative cystometric traces from control group with a mean of 4 ± 0.5 voids in 2 hours (Figure 10 D). The voided volume recorded in control rats was 2.75 ± 0.23 ml, and decreased to 0.75 ± 0.43 ml with PMA (Figure 10 B and C, N = 4, p ≤ 0.05 to control). PMA also caused a significant increase in the voiding frequency to 8 ± 0.9 voids over the same time course (Figure 10 D).

### Effects of PKC activation by PDBu on urinary bladder function in vivo evaluated by cystometry

**Figure 11:**
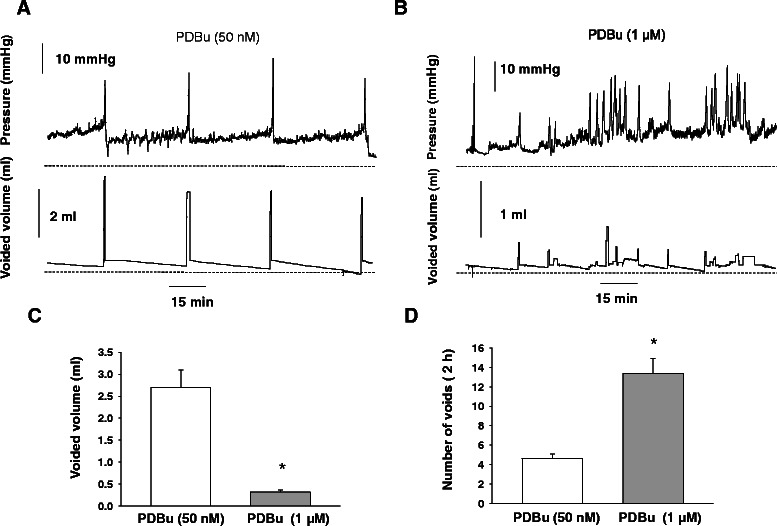
Urodynamic recordings from non-anesthetized rats upon intravesical instillation of low and high PDBu. **A**, Representative traces of intravesical pressure (top panel) and voided volume (lower panel) in rats instilled with low concentration of PDBu (50 nM). **B**, Representative traces of intravesical pressure (top panel) and voided volume (lower panel) in rats instilled with high concentration of PDBu (1 μM). **C**, Perfusion of the urinary bladder *in vivo* with low PDBu did not affect the voided volume **(C)** nor the number of voids **(D)**. However, high concentration of PDBu caused a significant increase in the frequency of micturition contractions (**B**; upper panel), and a decrease in voided volume (**B**; lower panel) in unanesthetized rats.

In order to determine if low PDBu affected micturition contractions, we tested the effects of intravesical instillation of low (50 nM) and high (1 μM) concentrations of PDBu in *in vivo* studies. Figure 11 shows the cystometric recordings upon intravesical instillation of low (A, 50 nM) and high (B, 1 μM) concentrations of PDBu on urodynamic parameters of rat urinary bladder. The data revealed that low concentration of PDBu had no significant effect on frequency of micturition (Figure 11D), nor affected the voided volume (Figure 11C), and was similar to control (Figure 10). However, high concentration of PDBu caused a significant increase in the frequency of micturition contractions (Figure 11B and D) along with a commensurate decrease in the volume of voided urine from 2.7 ± 0.4 ml to 0.3 ± 0.048 ml per cycle (Figure 11C, p ≤ 0.05).

### Intravesical instillation of Ro318220 decreases the micturition volume and increases the number of non-voiding contractions during cystometry

**Figure 12:**
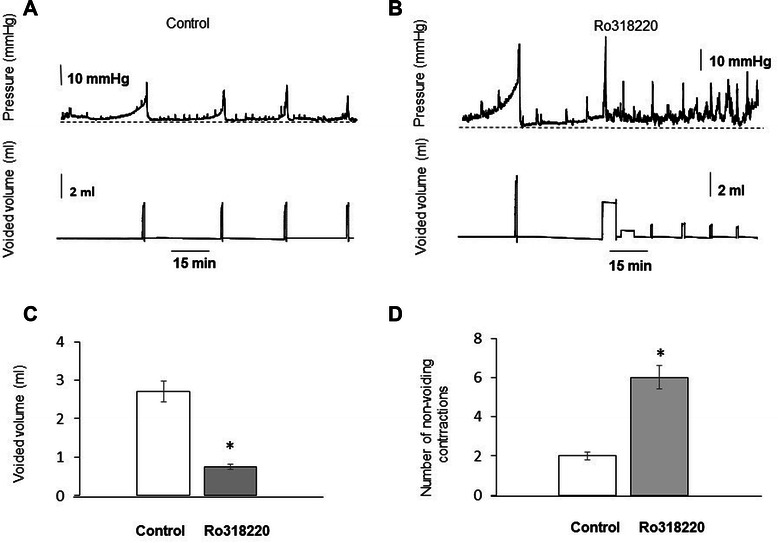
Urodynamic recordings showing the effect of PMA inhibitor, Ro318220, after intravesical instillation *in vivo.***A**, Representative traces of intravesical pressure (top panel) and voided volume (lower panel) in a control rat instilled with saline. **B**, Representative traces of intravesical pressure (top panel) and voided volume (lower panel) in a rat instilled with Ro318220 (50 μM). **C**, Changes in voided volume after instillation with Ro318220. **D**, Number of non-voiding contractions after intravesical application of Ro318220 was increased from **2** ± 0.5 to 6 ± 1.1 over 1 h period. The decrease in voided volume was followed by a two-fold increase in frequency over the same time period (N = 4, p ≤ 0.05 to control group).

Urodynamic evaluation of bladder function was performed in the presence of a second PKC inhibitor, Ro318220. Figure 12 shows cystometry traces recorded in control rat without Ro318220 treatment (A) and after intravesical instillation of the drug (B). The data revealed that Ro318220 application caused a 3-fold decrease in voided volume (Figure 12 C), and an increase in NVC from 2 ± 0.5 to 6 ± 1.1 (Figure 12 D, N = 4, p ≤ 0.05 to control).

### In vivo treatment with Bim-1 decreases the micturition volume and increases the number of non-voiding contractions during cystometry

**Figure 13:**
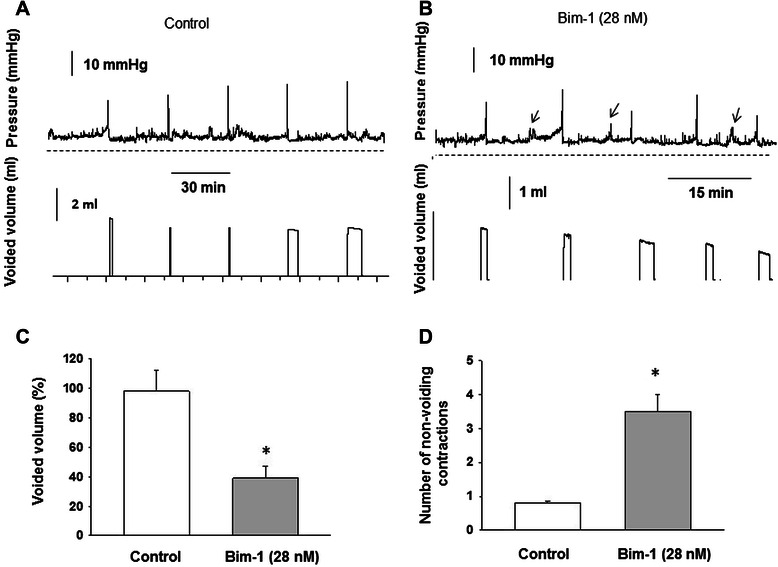
Inhibition of PKC activity by Bim-1 enhanced the number of non-voiding contractions recorded in vivo. **A**, Representative traces of intravesical pressure (top panel) and voided volume (lower panel) in a control rat instilled with saline. **B**, Representative traces of intravesical pressure (top panel) and voided volume (lower panel) in a rat instilled with intravesical Bim-1 (28 nM). Arrows point to non-voiding contractions. **C**, Changes in voided volume after pre-treatment with Bim-1. **D**, Number of non-voiding contractions after intravesical application of PKC inhibitor. Bim-1 decreased the voided volume by 56%, and increased the number of NVC 3.5-fold (p < 0.05, N = 4).

This set of experiments was designed to determine whether or not Bim-1, a PKC inhibitor, promotes NVC in the urinary bladder under *in vivo* conditions. Cystometrograms recorded in awake rats reflect the changes in urodynamic parameters during intravesical instillation of either saline (Figure 13 A) or Bim-1 (Figure 13 B). The upper panels of the figures represent the intravesical pressure, whereas the lower panels show the volumes of voided urine. Intravesical instillation of Bim-1 (28 nM) decreased the voided volume of urine by 56 ± 4.8% (Figure 13 C, N = 4, p ≤ 0.05), and increased the number of non-voiding contractions by 3.5-fold (Figure 13 D, N = 4, p ≤ 0.05 to control). An increase in the number of NVC was consistent with the increased spontaneous activity seen in isolated muscle strips in response to Bim-1 application [22]. The reduced emptying, on the other hand, may be due to the inhibitory effect of Bim-1 on the IF or force maintenance in DSM as demonstrated in Figure 4, and as previously established in the rabbit bladder [22] .

### Application of a low concentration of PDBu after intravesical application of Bim-1 rescues bladder function

**Figure 14:**
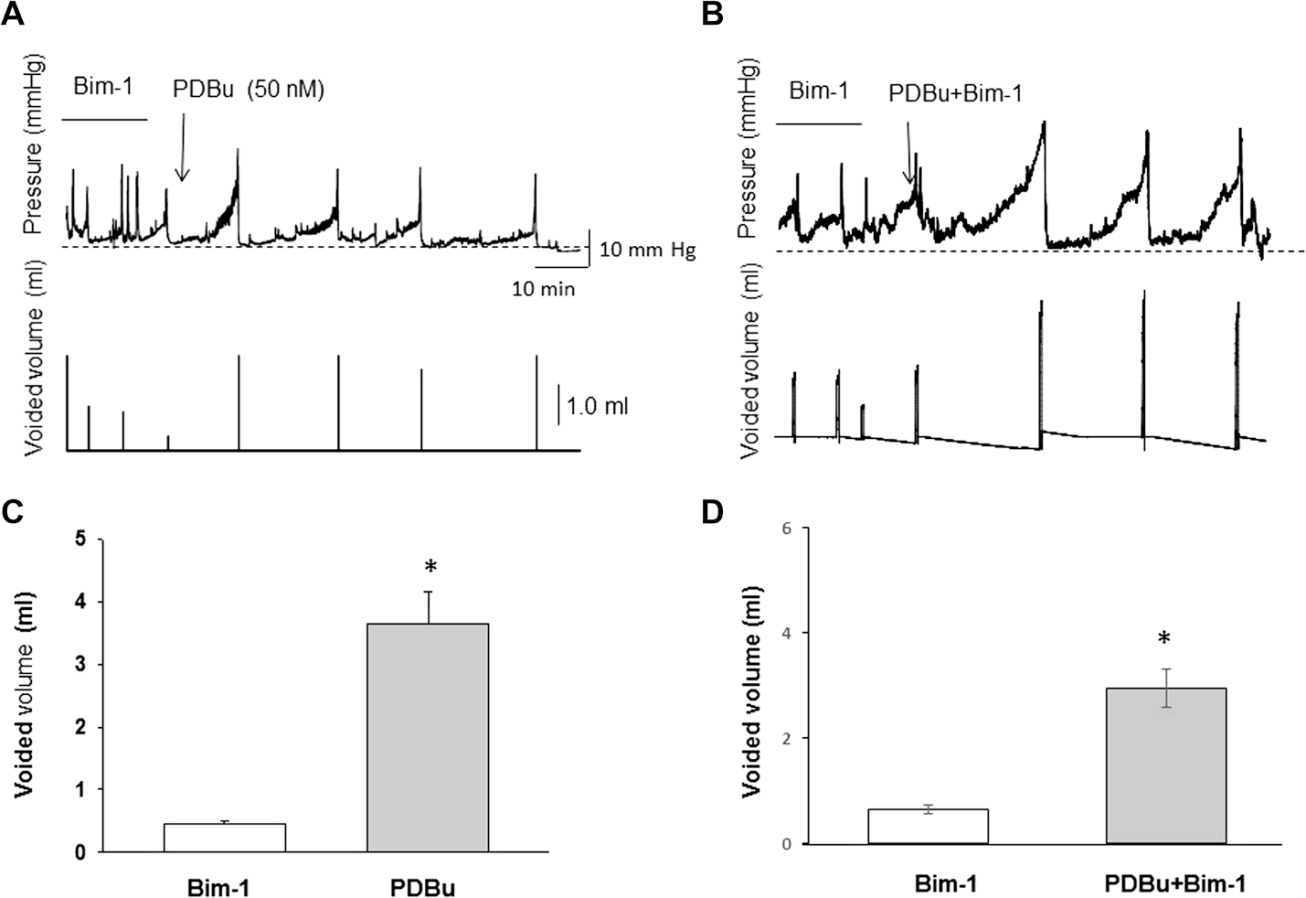
Intravesical PKC activation restores urodynamic parameters when applied after treatment with PKC inhibitor, Bim-1. **A**, Changes in intravesical pressure (top panel) and volume of voided urine (bottom panel) during bladder perfusion with Bim-1 (28 nM) followed by instillation of 50 nM of PDBu. Low concentration of PDBu reversed the effects of Bim-1 by suppressing non-voiding contractions and increasing the voided volume to control levels. **B,** In order to determine whether or not the recovery to control levels was due to a washout effect of the Bim-1 during PDBu instillation, we repeated these experiments maintaining the concentration of Bim-1 but increasing the PDBu concentration to 100 nM. The results confirmed the previous findings demonstrating that application of PDBu after intravesical instillation of Bim-1 rescues bladder function. **C,** Comparison of the voided volume changes upon Bim-1 and PDBu applications, and upon PDBu application in the presence of Bim-1 **(D)**.

In order to determine whether the effects of Bim-1 on bladder function can be reversed by subsequent application of the PKC activator, PDBu (50 nM) was infused following intravesical instillation of Bim-1 (Figure 14 A). The obtained results showed that a low concentration of PDBu inhibited Bim-1-induced non-voiding contractions in the urinary bladder, restored the duration of the micturition cycle, and increased the voided volume to control levels (Figure 14 C) when compared with the effects of Bim-1 (shown at the beginning of the tracing in Figure 14 A). Additional studies (Figure 14 B, and D) confirmed that these findings were not simply due to a washout effect of the Bim-1 during infusion of PDBu. In these experiments, we maintained the concentration of Bim-1 as in Figure 14 A, but had to increase the concentration of PDBu to 100 nM in order to see the same effect as in Figure 14 A. Since Bim-1 is a competitive inhibitor of PDBu, the higher concentration of PDBu was most likely able to overcome the effects of the initial Bim-1 infusion thus restoring a normal micturition cycle. These results confirm our *in vitro* findings and suggest that PKC down-regulation, *e.g.* as reported in PBOO [19], may contribute to frequency of micturition under pathophysiological conditions.

## Discussion

Physiological function of the urinary bladder is predicated on an increase in urine volume during the storage phase occurring at low intravesical pressure, and periodic elimination mediated by a rapid, phasic contraction that is maintained long enough to ensure complete bladder emptying [48-50]. These events are modulated by the neural input from the central nervous system (CNS) influencing detrusor-sphincter coordination [10]. Descending inhibitory inputs from the brain contribute to a relatively quiescent, and stable bladder smooth muscle (BSM) pressure during the storage phase, free of spontaneous and NVC that could increase BSM tone prematurely, raise intravesical pressure, and consequently reduce bladder storage capacity leading to voiding frequency. The mechanism by which this quiescent state is maintained and regulated during the micturition cycle, and the contribution of local mediators within the bladder wall, have not been adequately explored, and is not well understood.

A number of previous studies using animal and human bladders established that disruption of the quiescent state of the BSM leads to detrusor overactivity, and increased frequency of micturition [5,19,22,51]. It was also established that endogenous PKC displayed a stimulus- and concentration-dependent activation profile by inhibiting spontaneous myogenic contractions and BSM tone at low levels of activation, however, increasing BSM force at higher levels of activation [22]. Overall, these studies, along with current results, provide insights into how bladder pressure, voiding reflexes, and detrusor contractility are modulated by PKC during the micturition cycle.

In the present study, we provided direct evidence that PKC activation by high PDBu increases the sensitivity, and enhances the contractile responses of urothelium intact BSM strips to EFS *in vitro*. It was also reported that another PKC activator, PMA, can enhance the maximum response to EFS in urothelium denuded muscle strips of the guinea pig, in vitro [20]. These results are in line with previous reports which confirmed that phorbol esters can enhance the contractile response to EFS [20,52,53], however, these groups did not determine the sensitivity, and concentration-dependent relationship. High levels of PKC activation by PDBu increased the contractile responses of bladder strips to EFS by shifting the frequency response curve to the left (Figures 2 and 7), and increased the duration of muscle force maintenance which is essential for bladder emptying. *In vivo* cystometry studies established that enhanced PKC activation, by both PDBu and PMA, also increased the frequency of nerve-dependent micturition contractions, and decreased the voided volume (Figures 10 and 11). The ability of high levels of PKC stimulation to increase voiding frequency suggests that activation of endogenous PKC signaling may play a role in the relay of information from the filling urinary bladder to the CNS. For instance, several studies previously determined that PKC may participate in sensory mechanotransduction [38,54]. An increase in micturition frequency may be also due to a direct contractile effect of high concentration of PKC activator on the DSM, or the urothelium, both of which have been shown to express PKC, thereby, increasing intravesical pressure above the threshold required to initiate a micturition contraction. This is also in compliance with the fact that enhanced PKC activation by PDBu triggers an increase in BSM tone [19,22]. However, it is not known if PDBu can cross the mucosal barrier and penetrate into the muscle layer to cause a contraction of the BSM, or whether it affects the release of some intrinsic factors from the urothelium leading to contraction.

Our data also revealed opposing actions of high PDBu on EFS-induced frequency response *vs* carbachol-induced concentration responses in isolated BMS. High PDBu induced increased sensitivity of responses to EFS as previously mentioned. Prior studies have also shown that activation of PKC with phorbol esters can increase the release of neurotransmitters from nerve terminals in *in vitro* [46,54]. Both low (50 nM), and high PDBu (1 μM), on the other hand, decreased sensitivity to carbachol stimulation without effects on the PF. This data suggests that in the absence of direct nerve stimulation by EFS, PKC activation by PDBu may have a desensitizing effect on cholinergic sensitivity when the nerves are bypassed, and receptors are directly stimulated, as reported previously for muscarinic receptors [48]. We surmise that the ability of high, and low PKC stimulation to modulate cholinergic sensitivity may be relevant during the mid and later stages of the of the micturition cycle, since cholinergic activity is known to increase as the bladder expands [55,56]. Thus, PKC may play a role in insuring that the cholinergic response to bladder expansion is not too rapid or premature, increasing BSM tone, and restricting bladder storage capacity. This conclusion is supported by the fact that both PKC inhibitors, Bim-1 and Ro318220, induced an increase in NVC, decreased voided volumes, and increased frequency of urination during *in vivo* cystometry. The fact that inhibition of endogenous, or resting levels PKC activity by Bim-1 did not affect carbachol sensitivity in isolated muscle strips (Figure 9) may indicate that carbachol sensitivity is mediated by a stimulated increase of baseline PKC activity as the bladder expands. Similar results regarding the effects of Bim-1, on carbachol sensitivity, were obtained by Schneider et al. in the human detrusor in response to carbachol stimulation [57].

Endogenous PKC activity may play a role in maintaining the quiescent state of the bladder during storage by modulating the amplitude of spontaneous contractions in the detrusor [22]. This study demonstrated that low PKC stimulation by PDBu inhibited spontaneous myogenic contractions *in vitro.* Additional data provided evidence that down-regulation of PKC in PBOO model was associated with increased frequency of urination, and a higher amplitude of spontaneous contractions [19]. In the current experiments, we infused the PKC inhibitor, Bim-1, intravesically during continuous cystometry. Similar to the results obtained *in vitro*, this intervention resulted in a significant increase in the number of NVC, which is the *in vivo* correlate of spontaneous contractions recorded *in vitro*. Furthermore, low PKC activity induced by PDBu was able to competitively antagonize and suppress Bim-1-induced NVC, and restore normal frequency of micturition (Figure 14). The ability to translate *in vitro* muscle strip studies into *in vivo* (cystometry) indicates that PKC or similar molecules may have significant potential therapeutic value in the treatment of overactive bladder syndrome.

**Figure 15:**
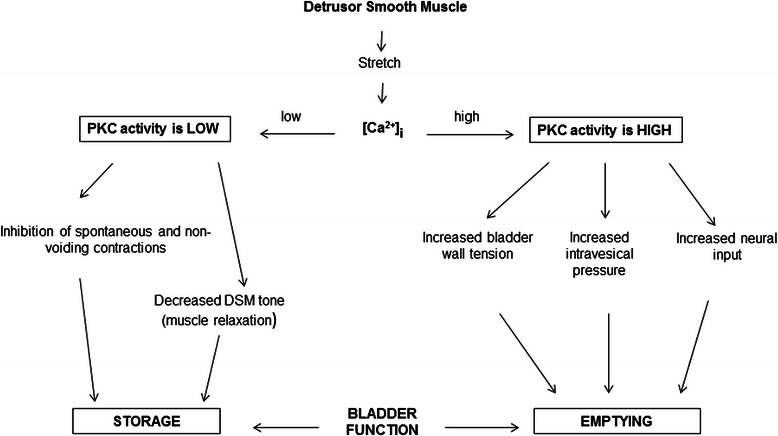
Schematic presentation of the potential mechanisms linking low and high PKC activity with detrusor smooth muscle tone, contractility and relaxation during bladder storage and emptying.

Presented data suggest that PKC modulates a wide range of integrative effects on urinary bladder function, including both storage and emptying, and acts via several mechanisms, affecting both bladder muscle and innervation in the bladder wall, in a concentration-dependent manner. One of the mechanisms associated with PKC regulation may involve stretch-induced release of intracellular calcium ([Ca ^2+^]) when the bladder expands during the storage phase [58,59]. Prior studies revealed that the bladder contains significant levels of the calcium-dependent PKC-alpha [19], and that stretch can result in the release of calcium from intracellular stores in bladder smooth muscle cells [58]. A low level of [Ca ^2+^]_i_ during the early storage phase would ensure low PKC activity which was shown to maintain the quiescent state of the urinary bladder by suppressing spontaneous and non-voiding contractions (Figure 15). Conversely, as the bladder increases in volume and approaches its capacity, both stretch, and stretch-induced calcium release may trigger an increase in calcium-dependent PKC activity, development of wall tension, enhanced PKC-dependent signaling, and, as a result, bladder emptying. This scenario is supported by our present results showing that low PKC stimulation inhibits spontaneous and non-voiding contractions under both *in vitro* and *in vivo* conditions, while high PKC stimulation increased BMS force, contractile response to EFS known to activate intrinsic nerves, and promoted BSM force maintenance (integral force).

Overall, our data suggest that the mechanisms of PKC action on bladder function involves complex interactions between smooth muscle and bladder nerves in a concentration dependent manner. We also confirmed that intrinsic bladder nerves coursing in the detrusor layer have to be stimulated in the presence of high PDBu in order to significantly increase BSM contractility at lower frequencies of EFS (Figure 2). This is in line with the results from the carbachol dose response curve showing decreased sensitivity to direct stimulation of muscarinic receptors (Figure 8). This data strongly indicates that the heightened sensitivity of the BMS in response to EFS is not a function of non-neuronal factors, but most likely due to increased release of neurotransmitters from nerve terminals upon EFS, as previously reported [54].

The complexity of the PKC effect on urinary bladder function is further demonstrated by our urodynamic studies showing that both inhibition of PKC by Bim-1, and activation of PKC by high concentration of PDBu induced frequency of micturition, and decreased voided volumes. We surmise that the underlying causes of these two actions are quite different. It was previously reported [22] and also confirmed in the present study, that inhibition of PKC by Bim-1 reduced the ability of DSM to maintain muscle force, but had no effect on sensitivity of the BMS to either the EFS frequency response (Figure 4) or carbachol-concentration response curves (Figure 8). Since endogenous, or resting levels of PKC activity are likely to be low in comparison to stimulated levels, it is possible that inhibition of these low levels of PKC did not affect EFS or carbachol sensitivity. This is consistent with our data showing that stimulated, higher levels of PKC activity affected the sensitivity to both EFS, and carbachol, causing an increased sensitivity to EFS, and a decreased sensitivity to carbachol. The current literature supports both of these actions since it was shown that PDBu can increase the release of neurotransmitters from nerve terminals, likely increasing sensitivity, while decreasing the response to direct muscarinic receptor stimulation [48].

One of the limitations of this study is that these complex interactions between smooth muscle and nerves in response to phorbol esters, exist on a continuum, and can complicate interpretation of the data, since it is not exactly clear where the smooth muscle effects end, and the nerve-mediated effects begin. Thus, it is possible that there may be some areas of overlap where the effects of smooth muscle *vs* neural effects have not been adequately characterized. However, since overactive bladder is a major concern of patients in clinical urology, the potential benefits of these pharmacological probes for contributing to the amelioration of clinical symptoms likely outweigh the negatives.

Thus, the similar outcomes of the inhibitor, and the activator of PKC on frequency, and voided volume may have different trigger points, and underlying causes. It is clear, and our data shows that high PDBu, and PMA increased the frequency of micturition contractions very early in the micturition cycle, which, by definition, are nerve-mediated. This invariably means that the void volume will be decreased as we have shown. The inhibitors of PKC, Bim-1 and ro318220, also resulted in a reduced void volume, while the number of micturition contractions stayed fairly constant over the same time course. This implies that the inhibitors *per se* did not increase micturition frequency over a similar period of time, but did reduce the void volume later in the micturition cycle compared to high PDBu and PMA. The activators demonstrated a fairly quick, and affirmative effect on voiding early in the micturition cycle, while the effect of the inhibitors was more progressive and delayed, suggesting that the later effects are rather secondary with slower kinetics. Thus, though not conclusive, it is more likely that the quick response to high PDBu and PMA early in the micturition cycle during cystometry, is mediated by effects on nerves, while the much slower response to the inhibitors is mediated by either effects on the urothelium or smooth muscle. In this regard, we have shown that both inhibitors reduced the ability to maintain muscle force, a requirement for efficient bladder emptying, and may be responsible for the decreased void volumes.

It is well established that bladder function is modulated by the descending input from the CNS. Our data, along with prior studies, suggests that local regulation by PKC may also play an important complementary role in both the inhibitory (storage), and stimulatory (emptying) phases of the micturition cycle. A decrease of descending inhibitory activity from the brain (*e.i.* “disinhibition”) to bladder motor neurons may lead to detrusor overactivity [10]. Similarly, our data from this, and prior studies suggests that a diminution of PKC activity within the bladder wall due to pharmacologic inhibition, or pathologic changes, may also lead to detrusor overactivity. Both nerve-mediated and smooth muscle-related mechanisms often complement each other, as it was shown that partial denervation of the detrusor may alter the properties of the smooth muscle via the spread of electrical signals between smooth muscle cells as established for myogenic detrusor overactivity [3]. Thus, because of its ability to modulate both nerve- and muscle-mediated contractility of the bladder in a dose-dependent manner, PKC could be one of the common links between the neurogenic and myogenic hypotheses of detrusor overactivity.

## Conclusions

We have provided evidence that PKC stimulation in the rat urinary bladder is involved in the regulation of bladder storage and emptying in a concentration-dependent manner. Our data suggest that PKC may also be involved in the modulation of both neuronal, and non-neuronal, bladder contractility. We have also shown that the underlying basis of this regulation involves the progressive and concentration-dependent activation profile of PKC which was shown to be comparable between *in vitro* and *in vivo* conditions. Present data are consistent with prior findings *in vitro*, and confirm a major physiological role for PKC in the regulation of bladder storage and emptying within the micturition cycle. Future studies are warranted to identify precise regulatory conditions modulating endogenous PKC activity in the urinary bladder and associated effects on the micturition cycle. This knowledge will provide a foundation for the development of novel therapeutic approaches to treat voiding dysfunction.

## Abbreviations

Bim-1: Bisindolylmaleimide1
BK: Large conductance calcium-activated potassium channel
BMS: Bladder muscle strip
BSM: Bladder smooth muscle
CNS: Central nervous system
DSM: Detrusor smooth muscle
EFS: Electrical field stimulation
IF: Integral force
MLCP: Myosin light chain phosphorylation
NVC: Non-voiding contractions
OAB: Overactive bladder
PBOO: Partial bladder outlet obstruction
PDBu: Phorbol-12,13-dibutyrate
PF: Peak force
PMA: Phorbol-12,13-myristate
ROK: Rho-associated kinase
